# Comparing the diagnostic efficacy of [^18^F]FDG PET/CT and [^18^F]FDG PET/MRI for detecting bone metastases in breast cancer: a meta-analysis

**DOI:** 10.2478/raon-2023-0037

**Published:** 2023-07-26

**Authors:** Longjie Xia, Jianqin Lai, Di Huang, Shenghui Qiu, Huiqiong Hu, Yunxiang Luo, Jie Cao

**Affiliations:** Department of General Surgery, The First Affiliated Hospital of Jinan University, Jinan University, Guangzhou, China; Department of General Surgery, Guangzhou First People's Hospital, Guangzhou, Guangzhou, China; Department of Plastic Surgery, The First Affiliated Hospital of Sun Yat-sen University, Guangzhou, China

**Keywords:** [^18^F]FDG PET/CT, [^18^F]FDG PET/MRI, bone metastases, breast cancer, meta-analysis

## Abstract

**Background:**

This meta-analysis aimed to evaluate the comparative diagnostic efficacy of [^18^F]FDG PET/CT and [^18^F] FDG PET/MRI in detecting bone metastases in breast cancer patients.

**Methods:**

An extensive search was conducted in the PubMed, Embase, Web of Science, and Cochrane Library databases to identify available publications up to February 2023. Studies were included if they evaluated the diagnostic efficacy of [^18^F]FDG PET/CT and [^18^F]FDG PET/MRI in patients with breast cancer bone metastases. Sensitivity and specificity were assessed using the DerSimonian and Laird method, followed by transformation via the Freeman-Tukey double inverse sine transformation.

**Results:**

16 articles (including 4 head-to-head comparison articles) involving 1,261 patients were included in the meta-analysis. The overall sensitivity of [^18^F]FDG PET/CT in patient-based analysis, lesion-based analysis, and head-to-head comparison were 0.73, 0.89, and 0.87, respectively, while the overall sensitivity of [^18^F]FDG PET/MRI were 0.99, 0.99, and 0.99. The results indicated that [^18^F]FDG PET/MRI appears to a higher sensitivity in comparison to [^18^F]FDG PET/CT(all *P* < 0.05). In contrast, the overall specificity of [^18^F]FDG PET/CT in patient-based analysis, lesion-based analysis, and head-to-head comparison were 1.00, 0.99, and 1.00, respectively, while the overall specificity of [^18^F]FDG PET/MRI were 1.00, 0.99, and 0.98. These results suggested that [^18^F]FDG PET/CT has a similar level of specificity compared to [^18^F]FDG PET/MRI.

**Conclusions:**

Our meta-analysis indicates that [^18^F]FDG PET/MRI demonstrates superior sensitivity and similar specificity to [^18^F]FDG PET/CT in detecting bone metastases in breast cancer patients. Further prospective research is required to confirm these findings and assess the clinical application of these techniques.

## Introduction

Breast cancer is a serious global health concern and is the most prevalent malignancy affecting women.^1^ Bone metastasis is a frequent complication of advanced breast cancer, with nearly 65% of patients developing bone metastases.^2^ The existence of bone metastases can cause severe morbidity and death, as well as reduced quality of life and an increased risk of skeletal-related events.^3^ Hence, early identification of bone metastases is essential for effective treatment strategies and improved patient outcomes. For the detection of bone metastases in breast cancer, conventional imaging techniques such as X-ray, bone scintigraphy, and computed tomography (CT) have been utilized.^4^ However, these modalities have limits in terms of sensitivity, specificity, and spatial resolution.^5^

With higher sensitivity and specificity, as well as the capacity to provide both metabolic and anatomical information, [^18^F]Fluorodeoxyglucose ([^18^F] FDG) positron emission tomography/computed tomography (PET/CT) and [^18^F]FDG positron emission tomography/magnetic resonance imaging (PET/MRI) have emerged as promising imaging modalities to identify bone metastases in breast cancer patients.^6^ [^18^F]FDG is a radiotracer that accumulates in cancer cells and can be detected via positron emission tomography (PET). PET/CT imaging combines PET and CT imaging to provides metabolic as well as anatomical information, whereas PET/MRI imaging combines PET and magnetic resonance imaging (MRI) for a more detailed analysis of soft tissue structures.^7,8^

Numerous studies have been conducted to assess the diagnostic accuracy of [^18^F]FDG PET/CT and [^18^F]FDG PET/MRI in detecting bone metastases in breast cancer patients, with inconsistent results. Some studies have shown that [^18^F]FDG PET/MRI is superior to [^18^F]FDG PET/CT in terms of sensitivity^9,10^, while others have reported similar diagnostic performance for both modalities.^11^

Therefore, a meta-analysis should be conducted to assess the diagnostic efficacy of [^18^F]FDG PET/CT and [^18^F]FDG PET/MRI for detecting bone metastases in breast cancer. The current meta-analysis would provide an overall comparison of the diagnostic efficacy of the two modalities, based on the extracted data from all available identified studies.

## Methods

The meta-analysis followed the Preferred Reporting Items for a Systematic Review and Meta-analysis of Diagnostic Test Accuracy (PRISMA-DTA) guidelines.^12^ The protocol of the current meta-analysis has been registered with PROSPERO (CRD42023402353).

### Search strategy

An extensive search was conducted in the PubMed, Embase, Web of Science, and Cochrane Library databases to identify available publications up to February 2023. The search was conducted using the following keyword terms: “Positron-Emission Tomography”, “Breast Neoplasms” and “Bone metastases”. More details could be found in the Supplementary Table 1. The reference lists of the included studies were manually searched to find additional relevant articles.

**TABLE 1.** Study and patient characteristics of the included studies for [^18^F]FDG PET/CT

**Author**	**Year**	**Type of imaging test**	**Study characteristics**					**Patient characteristics**		

			**Country**	**Study design**	**Analysis**	**Reference standard**	**No. of patients**	**Clinical indication**	**Mean/Median age**	**Previous treatment**
Catalano *et al*.^15^	2015	PET/CT	Italy	Retro	PB	Pathology and/or follow-up imaging	109	Initial stage and post-treatment stage	Mean ± SD: (58.08 ± 10.7)	Surgery
Melsaether *et al*.^10^	2016	PET/CT	USA	Pro	LB	Pathology and/or follow-up imaging	51	Initial stage and post-treatment stage	Mean(range): 56 (32–76)	Chemotherapy
Botsikas *et al*.^9^	2018	PET/CT	Switzerland	Pro	PB and LB	Pathology and/or follow-up imaging	80	Initial stage and post-treatment stage	Mean ± SD: (48 ± 12.9)	NA
Sawicki *et al*.^11^	2016	PET/CT	Germany	Pro	LB	Pathology and/or follow-up imaging	21	Post-treatment stage	Mean ± SD: (59.4 ±11.5)	NA
Balci *et al*.^17^	2012	PET/CT	Turkey	Retro	PB	Pathology and/or follow-up imaging	162	Initial stage and post-treatment stage	Mean: 50.6	Surgery
Hahn *et al*.^18^	2011	PET/CT	Germany	Retro	PB and LB	Follow-up imaging	29	Initial stage	Mean (range): 57.5 (35–78)	NA
Manohar *et al*.^19^	2012	PET/CT	India	Retro	LB	Pathology and/or follow-up imaging	111	Post-treatment stage	Mean(range): 52 (22–80)	Surgery
Niikura *et al*.^25^	2011	PET/CT	Japan	Retro	LB	Pathology and/or follow-up imaging	225	Initial stage and post-treatment stage	Mean: 53.4	Chemotherapy or endocrine therapy
Riegger *et al.*^22^	2012	PET/CT	Germany	Retro	LB	Pathology and/or follow-up imaging	106	Initial stage	Mean ± SD: (57 ± 13)	NA
Rager *et al*.^23^	2018	PET/CT	Switzerland	Retro	PB and LB	Follow-up imaging	25	Initial stage and post-treatment stage	Median(range): 5 (38–82)	NA
Demir *et al.*^20^	2014	PET/CT	Turkey	Retro	LB	Pathology and/or follow-up imaging	50	Post-treatment stage	Mean ± SD: (53.9 ± 12.3)	NA
Hansen *et al*.^24^	2015	PET/CT	Denmark	Pro	LB	Pathology	18	Post-treatment stage	Mean(range): 61.5 (38–76)	Surgery
Niikura *et al*.^21^	2016	PET/CT	Japan	Pro	PB	Pathology and/or follow-up imaging	28	Initial stage and post-treatment stage	Median(range): 59 (31–76)	Surgery
Shawky *et al.*^26^	2016	PET/CT	Egypt	Pro	LB	Pathology and/or follow-up imaging	30	Post-treatment stage	Mean(range): 53.5 (33–73)	Surgery or Chemotherapy or radiotherapy
Teke *et al.*^27^	2020	PET/CT	Turkey	Retro	LB	Follow-up imaging	62	Initial stage	Median(range): 44.5 (8–81)	NO

LB = lesion-based; NA = not available; PB = patient-based; Pro = prospective; Retro = retrospective

### Inclusion and exclusion criteria

Studies were included in this meta-analysis if they evaluated the diagnostic performance of [^18^F]FDG PET/CT and/or [^18^F]FDG PET/MRI in patients with breast cancer bone metastases with a sample size of more than 10 patients.

Duplicated articles, abstracts without full texts, editorial comments, letters, case reports, reviews, meta-analyses, irrelevant titles and abstracts, and non-English full-text articles were excluded. Studies with incomplete or unclear data necessary to calculate the sensitivity or specificity of the imaging modality being studied were excluded. In addition, studies using PET without CT or MRI, or using different radiotracers were excluded. For studies using the same data, only the latest studies were taken into consideration.

### Retrieval of relevant articles

Two researchers independently read the titles and abstracts of retrieved articles using the predetermined selection criteria. Subsequently, full-text evaluation was conducted to ascertain each study's eligibility. The event of discrepancies between the researchers were resolved through discussion, ultimately arriving at a consensus.

### Quality assessment

Two researchers independently assessed the quality of the included studies utilizing the Quality Assessment of Diagnostic Performance Studies (QUADAS-2) tool.^13^ The QUADAS-2 tool encompasses four essential domains: (1) patient selection; (2) index test; (3) reference standard; and (4) flow and timing. The risk of bias was rated as “high risk,” “low risk,” or “unclear risk.”

In assessing the risk of bias, several key aspects were evaluated. First, patient selection bias was addressed by enrolling consecutive patients. Second, the results of the index test were evaluated independently of the outcomes of the reference standard to minimize potential bias. Third, the reference standard was evaluated without knowledge of the results of the index test to ensure objectivity. Finally, the flow and timing aspect examined the appropriateness interval (less than 3 months) between the index tests and the reference standard. Regarding applicability concerns, the analysis focused on three main questions. First, patient selection was “Are there any concerns regarding the relevance of the included patients to the scope of the review?” Second, the index test was “Are there concerns that the target condition as defined by the reference?” Third, the reference standard was “Are there concerns about the compatibility between the target condition, as established by the reference standard, and the review question?”

### Data extraction

Two researchers independently extracted data from all the included articles. The gathered data included information about the author, year of publication, and the type of imaging test used in the study, study features (country, study design, analysis, and reference standard), characteristics of patients (number of patients, clinical indication, mean/median age, and previous treatment), and technical aspects (scanner modality, ligand dose, and image analysis).

In cases of disagreements, the researchers discussed the issue until a consensus was reached to ensure accuracy in the extracted data.

### Outcome measures

The main outcome measure were the sensitivities and specificities of [^18^F]FDG PET/CT and [^18^F]FDG PET/MRI in patient-based analysis, lesion-based analysis and head-to-head comparison. Sensitivity was defined as the ratio of patients or lesions with true positive (TP) scans to the sum of TP and false negative (FN) scans for either patients or lesions have been reported; Specificity was defined as the ratio of patients or lesions with true negative (TN) scans to the sum of TN scans and false negative (FN) scans have been reported.

### Statistical analysis

Sensitivity and specificity were assessed using the DerSimonian and Laird method, followed by transformation via the Freeman-Tukey double inverse sine transformation. The Jackson method was used to calculate the confidence intervals. The Cochrane Q and I^2^ statistics were used to assess the heterogeneity within and between groups.^14^ If the heterogeneity between the studies differed significantly (*P* < 0.10 or I^2^ > 50%), sensitivity analysis was performed by reassessing the sensitivities or specificities following the omission of articles one by one. This was done to evaluate the robustness of the overall sensitivities or specificities and to identify single studies that may contribute to heterogeneity.

We evaluated publication bias by employing both funnel plot and Egger's test (for outcomes including over 10 studies). For all statistical tests except heterogeneity (*P* < 0.10), a significance level of *P* < 0.05 was considered statistically significant. Statistical analyses were conducted using R software version 4.1.2 for statistical computing and graphics.

## Results

### Search strategy and study selection

**FIGURE 1.**
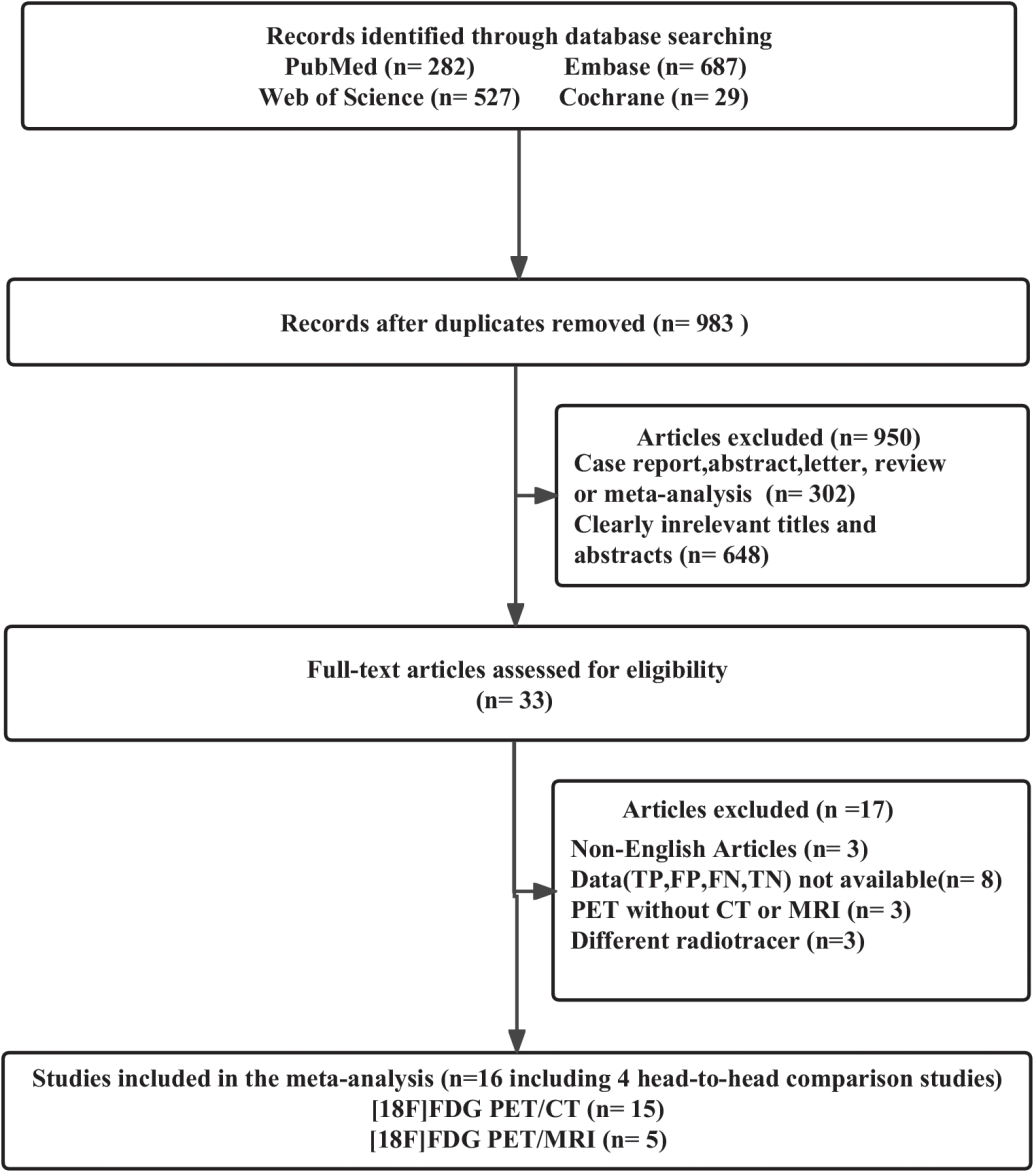
PRISMA flow diagram illustrating the study selection process. FN = false negative; FP = false positive; TN = true negative; TP = true positive

The preliminary search revealed a total of 1525 publications. However, 542 studies were considered duplicates, and another 950 did not meet the eligibility criteria and were therefore not included in the study. After a comprehensive review of the full texts of the remaining 33 articles, another 17 were deemed ineligible for the study either because data (TP, FP, FN, and TN) were not available (n = 8) or the radiotracer was different (n = 3). In addition, non-English articles (n = 2) and PET without CT or MRI articles (n = 3) were excluded. Finally, 16 articles^9,10,11,15,16,17,18,19,20,21,22,23,24,25,26,27^ (including 4 head-to-head comparison articles) evaluating the diagnostic efficacy of [^18^F]FDG PET/CT (n = 15)^17,18,19,20,22,23,24,25,26,27^, and [^18^F] FDG PET/MRI (n = 5)^9,10,11,15,16^ were included in the meta-analysis. The article selection process, according to the PRISMA flow diagram, is depicted in Figure 1.

### Study description and quality assessment

The 16 eligible studies included a total of 1,261 breast cancer patients (range from 18 to 225). Among the included studies, 9 articles were retrospective studies, while 7 articles were prospective studies. In terms of analysis methods, 3 articles employed patient-based analysis, 9 articles used lesion-based analysis, and 4 articles utilized both methods. 2 articles used pathology as the reference standard, 11 articles employed pathology and/or follow-up imaging as the reference standard, and 3 articles solely relied on follow-up imaging as the reference standard. Regarding clinical indications, 3 articles involved patients exclusively at the initial stage, 6 articles included patients only at the post-treatment stage, and the remaining 7 articles included patients at both initial and post-treatment stages. Table 1 and Table 2 summarize the study and patient characteristics of [^18^F]FDG PET/CT and [^18^F]FDG PET/MRI, while Supplementary Table 2 and Supplementary Table 3 present the technical aspects.

**TABLE 2.** Study and patient characteristics of the included studies for [^18^F]FDG PET/MRI

**Author**	**Year**	**Type of imaging test**	**Study characteristics**				**Patient characteristics**			

			**Country**	**Study design**	**Analysis**	**Reference standard**	**No. of patients**	**Clinical indication**	**Mean/Median age**	**Previous treatment**
Catalano *et al.*^15^	2015	PET/MRI	Italy	Retro	PB	Pathology and/or follow-up imaging	109	Initial stage and post-treatment stage	Mean ± SD: (58.08 ± 10.7)	Surgery
Bruckmann *et al.*^21^	2021	PET/MRI	Germany	Pro	PB and LB	Pathology	154	Post-treatment stage	Mean ± SD: (53.8±11.9)	NO
Melsaether *et al.*^10^	2016	PET/MRI	USA	Pro	LB	Pathology and/or follow-up imaging	51	Initial stage and post-treatment stage	Mean(range): 56(32–76)	Chemotherapy
Botsikas *et al.*^9^	2018	PET/MRI	Switzerland	Pro	PB and LB	Pathology and/or follow-up imaging	80	Initial stage and post-treatment stage	Mean ± SD: (48 ± 12.9)	NA
Sawicki *et al.*^11^	2016	PET/MRI	Germany	Pro	LB	Pathology and/or follow-up imaging	21	Post-treatment stage	Mean ± SD: (59.4 ± 11.5)	NA

LB = lesion-based; NA = not available; PB = patient-based; Pro = prospective; Retro = retrospective

**FIGURE 2.**
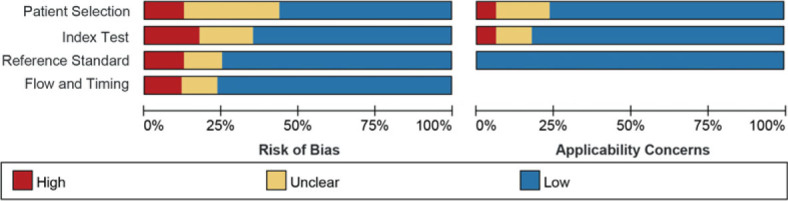
Risk of bias and applicability concerns of the included studies using the Quality Assessment of Diagnostic Performance Studies QUADAS-2 tool.

The risk of bias for each study according to the QUADAS-2 tool is illustrated in Figure 2. For the patient selection risk of bias assessment, we found 2 studies that graded as “high risk” since they didn’t include consecutive patients. For the index test, 3 studies were graded as “high risk” since the applied cut-off values were not pre-determined. With regards to the reference standard, 2 studies were graded as “high risk” as the final diagnosis was not determined independently by two or more physicians. The flow and timing standard were graded as “high risk” in 2 studies because some participants were excluded from data analyses. There were no major concerns with the quality of the included studies based on the overall quality assessment.

### Comparing the sensitivity of [^18^F] FDG PET/CT and [^18^F]FDG PET/MRI for detecting bone metastases in breast cancer

**FIGURE 3.**
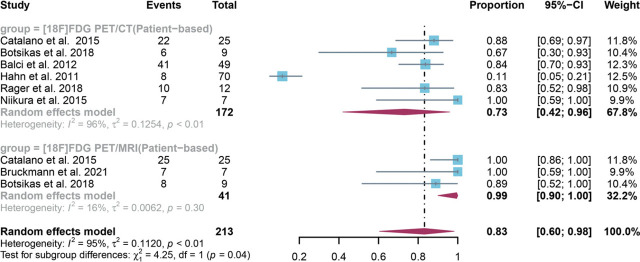
Forest plot showing the pooled sensitivities of [^18^F]FDG PET/CT and [^18^F]FDG PET/MRI in bone metastasis of breast cancer patients on a patient-based analysis. The plot displays individual study estimates (squares) with corresponding 95% confidence intervals (horizontal lines) and the pooled sensitivity estimate (diamond) for both modalities. The size of the squares represents the relative weight of each study in the meta-analysis.^9,15,17,18,21,23,25^

For patient-based analysis, a total of 8 studies with 213 patients were included in the analysis, and the pooled sensitivity of [^18^F]FDG PET/CT in detecting bone metastases in breast cancer was 0.73 (95% CI: 0.42–0.96), whereas [^18^F]FDG PET/MRI had an overall sensitivity of 0.99 (95% CI:0.90–1.00) (Figure 3). There was significant difference between [^18^F]FDG PET/CT and [^18^F]FDG PET/MRI in the sensitivity (*P* = 0.04) (Figure 3). After removing Hahn *et al.*'s study^18^ in our sensitivity analysis, the I^2^ value became 0%, suggesting it may be the potential source of heterogeneity. However, the results from the sensitivity analysis remained stable, with only minor variations observed, ranging from 0.66 to 0.86 (Supplementary Figure 1).

**FIGURE 4.**
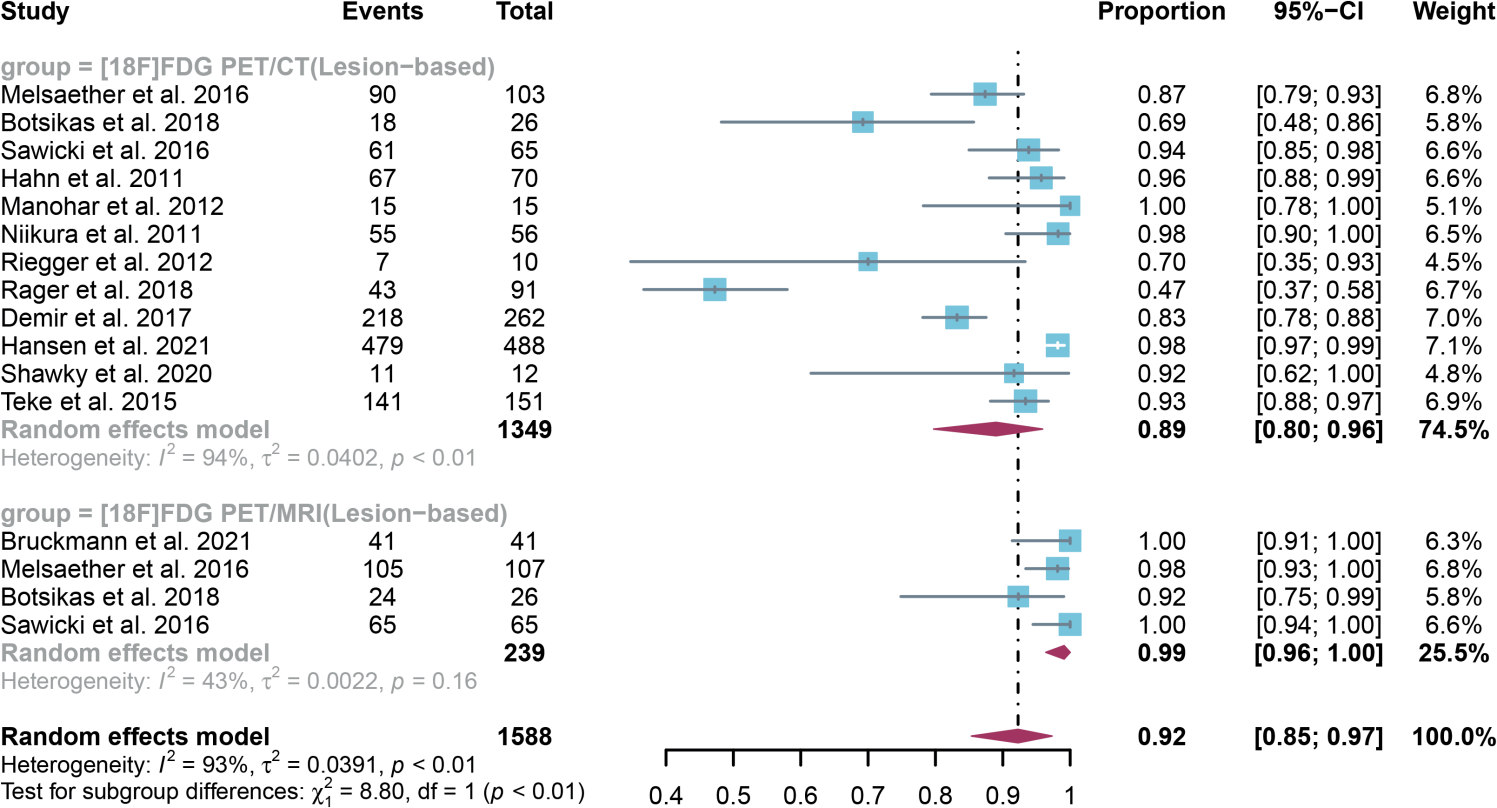
Forest plot showing the pooled sensitivities of [^18^F]FDG PET/CT and [^18^F]FDG PET/MRI in bone metastasis of breast cancer patients on a lesion-based analysis. The plot displays individual study estimates (squares) with corresponding 95% confidence intervals (horizontal lines) and the pooled sensitivity estimate (diamond) for both modalities. The size of the squares represents the relative weight of each study in the meta-analysis.^9,10,11,18,19,20,22,23,24,25,26,27^

For lesion-based analysis, a total of 13 studies with 1588 lesions were included in the analysis, and the pooled sensitivity of [^18^F]FDG PET/CT in detecting bone metastases in breast cancer was 0.89 (95% CI: 0.80–0.96), whereas [^18^F]FDG PET/MRI had an overall sensitivity of 0.99 (95% CI:0.96–1.00) (Figure 4). There was significant difference between [^18^F]FDG PET/CT and [^18^F]FDG PET/MRI in the overall sensitivity (*P* < 0.01) (Figure 4). Regarding the pooled overall sensitivity of [^18^F] FDG PET/CT in lesion-based analysis, the I^2^ was 94%. The sensitivity analysis revealed no potential source of heterogeneity. The results following sensitivity analysis remained stable, and only minor variations in the results ranging from 0.88 to 0.92 were noted (Supplementary Figure 2). The funnel plot and Egger's test revealed no evidence of publication bias for [^18^F]FDG PET/CT in lesion-based analysis (*P* = 0.30) (Supplementary Figure 3).

For head-to-head comparison, a total of 4 studies with 442 patients or lesions were included in the analysis, and the pooled sensitivity of [^18^F]FDG PET/CT in detecting bone metastases in breast cancer was 0.87 (95% CI: 0.77–0.94), whereas [^18^F]FDG PET/MRI had an overall sensitivity of 0.99 (95% CI:0.96–1.00) (Supplementary Figure 4). A significant difference was observed in the overall sensitivity between [^18^F]FDG PET/CT and [^18^F] FDG PET/MRI. (*P* < 0.01) (Supplementary Figure 4). Regarding the pooled overall sensitivity of [^18^F]FDG PET/CT in lesion-based analysis, the I^2^ was 64%. After removing Botsikas *et al*.'s study^9^ in our sensitivity analysis, the I^2^ value became 0%, suggesting it may be the potential source of heterogeneity. However, the results from the sensitivity analysis remained stable, with only minor variations observed, ranging from 0.83 to 0.90. (Supplementary Figure 5).

### Comparing the specificity of [^18^F] FDG PET/CT and [^18^F]FDG PET/MRI for detecting bone metastases in breast cancer

**FIGURE 5.**
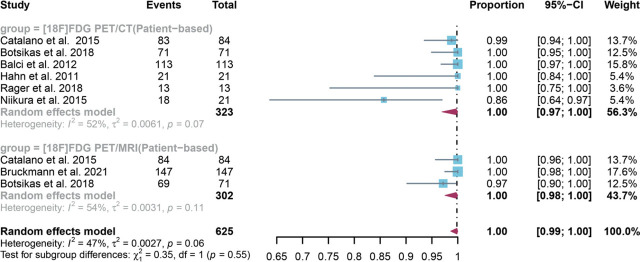
Forest plot showing the pooled specificities of [^18^F]FDG PET/CT and [^18^F]FDG PET/MRI in bone metastasis of breast cancer patients on a patient-based analysis. The plot displays individual study estimates (squares) with corresponding 95% confidence intervals (horizontal lines) and the pooled specificity estimate (diamond) for both modalities. The size of the squares represents the relative weight of each study in the meta-analysis.^9,15,17,18,21,23,25^

For patient-based analysis, a total of 7 studies with 625 patients were included in the analysis, and the pooled specificity of [^18^F]FDG PET/CT in detecting bone metastases in breast cancer was 1.00 (95% CI: 0.97–1.00), whereas [^18^F]FDG PET/MRI had an overall specificity of 1.00 (95% CI:0.98–1.00) (Figure 5). There was no significant difference between [^18^F]FDG PET/CT and [^18^F]FDG PET/MRI in the overall specificity (*P* = 0.55) (Figure 5). The pooled overall specificity of [^18^F]FDG PET/CT and PET/MRI exhibited I^2^ values of 52% and 54%, respectively. Sensitivity analysis revealed that removing Niikura *et al*.'s study^21^ reduced PET/CT heterogeneity (I^2^ = 0%), while removing Botsikas *et al*.'s study^9^ had a similar effect on PET/MRI. Nonetheless, both analyses yielded stable results, with minor variations between 0.99 and 1.00 (Supplementary Figures 6 and 7).

**FIGURE 6.**
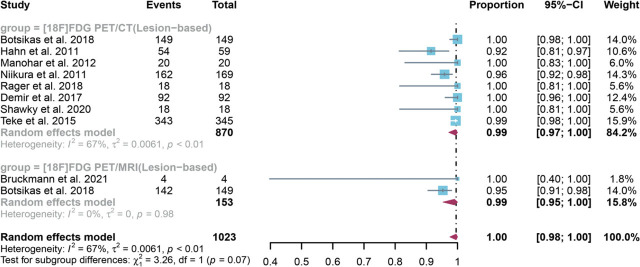
Forest plot showing the pooled specificities of [^18^F]FDG PET/CT and [^18^F]FDG PET/MRI in bone metastasis of breast cancer patients on a lesion-based analysis. The plot displays individual study estimates (squares) with corresponding 95% confidence intervals (horizontal lines) and the pooled specificity estimate (diamond) for both modalities. The size of the squares represents the relative weight of each study in the meta-analysis.^9,18,19,20,21,23,25,26,27^

For lesion-based analysis, a total of 9 studies with 1023 lesions were included in the analysis, and the pooled specificity of [^18^F]FDG PET/CT in detecting bone metastases in breast cancer was 0.99 (95% CI: 0.97–1.00), whereas [^18^F]FDG PET/MRI had an overall specificity of 0.99 (95% CI:0.95–1.00) (Figure 6). A significant difference was observed in the overall specificity between [^18^F]FDG PET/CT and [^18^F]FDG PET/MRI (*P* = 0.07) (Figure 6). Regarding the pooled overall specificity of [^18^F]FDG PET/CT in lesion–based analysis, the I^2^ was 67%. After removing Hahn *et al.*'s study^18^ in our sensitivity analysis, the I^2^ value became 49%, suggesting it may be the potential source of heterogeneity. However, the results from the sensitivity analysis remained stable, with only minor variations observed, ranging from 0.99 to 1.00. (Supplementary Figure 8).

For head-to-head comparison, a total of 2 studies with 466 patients or lesions were included in the analysis, and the pooled specificity of [^18^F]FDG PET/CT in detecting bone metastases in breast cancer was 1.00 (95% CI: 0.98–1.00), whereas [^18^F]FDG PET/MRI had an overall specificity of 0.98 (95% CI:0.91–1.00) (Supplementary Figure 9). No significant difference was observed in the overall specificity between [^18^F]FDG PET/CT and [^18^F]FDG PET/MRI (*P* = 0.50) (Supplementary Figure 9).

### Complementary role in identifying bone metastases of PET/CT, PET/MRI, MRI_(PET/MRI)_ alone, and CT_(PET/CT)_ alone for detecting bone metastases in breast cancer

In the 4 head-to-head comparison studies, one study (Melsaether *et al*.) did not provide information regarding the complementary role of PET/CT and PET/MRI in identifying bone metastases.^10^ Therefore, the evaluation was from 3 studies (Supplementary Table 4). Among these studies, PET/MRI correctly identified bone metastases in 10 out of 98 patients or lesions (10.2%) with initially negative PET/CT results. Conversely, PET/CT correctly identified bone metastases in none of the 86 patients (0%) with initially negative PET/MRI results.

Furthermore, 2 studies reported information on the detection of bone metastases in breast cancer patients using MRI_(PET/MRI)_ alone, while 4 studies provided data on CT_(PET/CT)_ alone. The results indicate that MRI_(PET/MRI)_ alone demonstrated a higher detection rate (65.5%, 180 out of 275) compared to CT_(PET/CT)_ alone (51.2%, 166 out of 324) (Supplementary Table 4).

## Discussion

In the field of detecting bone metastases in breast cancer, there has been uncertainty and controversy regarding the comparative diagnostic efficacy of [^18^F]FDG PET/CT and [^18^F]FDG PET/MRI.^9,15^ Key issues of comparison between the two imaging modalities include differences in sensitivity and specificity, as well as potential variations in diagnostic performance across different patient populations and analysis methods. To our knowledge, this is the first meta-analysis conducted on this topic, with patient-based, lesion-based and head-to-head comparison analysis, to compare the diagnostic efficacy of [^18^F]FDG PET/CT and [^18^F]FDG PET/MRI in detection of bone metastases in breast cancer patients.

The pooled sensitivity of [^18^F]FDG PET/CT in patient-based analysis, lesion-based analysis and head-to-head comparison were 0.73,0.89 and 0.87, while the pooled sensitivity of [^18^F]FDG PET/MRI were 0.99,0.99 and 0.99. In comparison to [^18^F]FDG PET/CT, it was suggested that [^18^F]FDG PET/MRI appeared to have a higher sensitivity (all *P* 0.05). In contrast, the pooled specificity of [^18^F]FDG PET/CT in patient-based analysis, lesion-based analysis and head-to-head comparison were 1.00,0.99 and 1.00, while the pooled specificity of [^18^F]FDG PET/MRI were 1.00,0.99 and 0.98. These findings indicated that [^18^F]FDG PET/CT and [^18^F]FDG PET/MRI have comparable levels of specificity.

Our results are in line with previous researches that have also suggested that [^18^F]FDG PET/MRI may have a higher sensitivity for detecting bone metastases compared to [^18^F]FDG PET/CT.^28^ In 2023, Zhang *et al*.^28^ conducted a meta-analysis to compare the diagnostic accuracy of [^18^F]FDG PET/CT and PET/MRI for detecting distant metastases in patients with various types of cancer. In the subgroup analysis including 3 studies of breast cancer (182 patients), they found that [^18^F]FDG PET/MRI demonstrated higher sensitivity (0.95 versus 0.87) and specificity (0.96 versus 0.94) compared to PET/CT. Our meta-analysis included a larger number of studies (16 studies) and patients than Zhang's study, which allowed us to perform a more comprehensive and robust analysis of the diagnostic efficacy of the two imaging modalities. Despite the difference, our study also provide evidence that PET/MRI has higher sensitivity and similar specificity compared to PET/CT in detecting bone metastases of breast cancer by adding more studies.

In 2019, Evangelista *et al*.^6^ conducted a head-to-head comparison study of [^18^F]FDG PET/CT and [^18^F]FDG PET/MRI for the evaluation of breast cancer. The authors included two head-to-head comparison studies that specifically focused on the detection of bone metastases in breast cancer. They reported that PET/MRI was able to detect more primary and skeletal/non-skeletal distant metastases compared to PET/CT. Our study and the study by Evangelista *et al*. are consistent in demonstrating the potential advantages of PET/MRI over PET/CT for the evaluation of bone metastasis in breast cancer. The superior sensitivity of [^18^F]FDG PET/MRI may be attributed to its capacity to provide both anatomical and functional information, which may be useful in cases where there is soft tissue involvement or bone marrow invasion.^29^

While the current meta-analysis found that [^18^F]FDG PET/MRI had a higher sensitivity than [^18^F]FDG PET/CT, it is important to note that [^18^F]FDG PET/MRI may not be available in all medical centers. The availability of [^18^F]FDG PET/MRI may also be affected by the medical center's location and resources. PET/CT provides high-resolution anatomical images and functional information from the PET component. In addition, it also has lower economic cost requirements compared to PET/MRI, making it a widely used imaging technique in clinical practice.^30,31^ PET/CT, on the other hand, has some limitations. One of the main limitations is the exposure to ionizing radiation, especially for younger patients or those who need repeated imaging exams.^10^

Overall, [^18^F]FDG PET/CT and [^18^F]FDG PET/MRI are both useful imaging modalities for detecting bone metastases in breast cancer patients, each having their own set of benefits and limitations. The choice of which imaging modality to use will depend on various factors such as the clinical situation, the accessibility of the imaging technique, and the preferences of the physicians.

In addition, another valuable diagnostic modality, Whole-body MRI (WB-MRI), also has demonstrated it capabilities. WB-MRI provides a comprehensive evaluation of the entire body with high sensitivity and excellent soft tissue contrast.^32^ On the other hand, PET/MRI combines functional and anatomical information, leading to improved specificity and simultaneous examination.^33^ To make a more accurate conclusion regarding the optimal tool for detecting bone metastasis, further head-to-head studies directly comparing WB-MRI and PET/MRI are needed.

Some limitations of the current meta-analysis should be considered when interpreting the results. Firstly, the heterogeneity of the included studies may have affected the overall sensitivities or specificities of [^18^F]FDG PET/CT and [^18^F]FDG PET/MRI, which may cause by different patient populations or imaging protocols. We therefore try to find out the source of heterogeneity by performing sensitivity analysis. Secondly, the studies included in the meta-analysis were mostly retrospective (9 of 16), which may have introduced bias. Third, pathology was not available for all lesions and patients, imaging follow-up was also used as the reference standard in cases where pathological examination was unavailable. Therefore, well-designed prospective studies with standardized imaging protocols and comprehensive pathological data are needed to confirm the findings of this meta-analysis.

## Conclusions

Based on the pooled results, our meta-analysis suggests that [^18^F]FDG PET/MRI has a higher sensitivity and similar specificity compared to [^18^F]FDG PET/CT in detection of bone metastases in breast cancer patients. Clinicians should consider the advantages and limitations of each imaging technique when making decisions about which method to use. Further studies with standardized imaging protocols and comprehensive pathological data are needed to confirm these findings and to explore the clinical utility of these imaging techniques.

## Supplementary Material

Supplementary Material Details
