# Supplementary material for: Comparing the diagnostic efficacy of [18F]FDG PET/CT and [18F]FDG PET/MRI for detecting bone metastases in breast cancer: a meta-analysis
Source: Radiol Oncol. 2023 Jul 26;57(3):299–309. doi: 10.2478/raon-2023-0037 (PMC10561067; doi:10.2478/raon-2023-0037)
Supplement: Supplementary file 1 — Supplementary Material Details [file raon-2023-0037_sm.pdf]

Comparing the diagnostic efficacy of [^{18}F]FDG PET/CT and [^{18}F]FDG PET/MRI for detecting bone metastases in breast cancer: a meta-analysis

Longjie Xia, Jianqin Lai, Di Huang, Shenghui Qiu, Huiqiong Hu, Yunxiang Luo, Jie Cao

Radiol Oncol 2023; 57(3): 299-309.; 57(3): 299-309.

doi: 10.2478/raon-2023-0037

SUPPLEMENTARY TABLE 1. Search strategy in *PubMed, Embase, Web of Science* and *Cochrane Library*

Database	Search strategy
PubMed (282)	("Breast Neoplasms"[Mesh] OR "Breast Neoplasm"[Title/Abstract] OR "Breast Tumor"[Title/Abstract] OR "Breast Cancer" [Title/Abstract] OR "Breast Carcinoma" [Title/Abstract] OR "Mammary Cancer"[Title/Abstract] OR "Mammary Carcinoma" [Title/Abstract] OR "Mammary Neoplasm"[Title/Abstract]) AND ("Positron-Emission Tomography"[Mesh] OR "PET"[Title/Abstract] OR "Positron-Emission Tomography"[Title/Abstract]) AND ("Bone metastasis"[Title/Abstract] OR "Bone metastases"[Title/Abstract])
Embase (687)	('breast tumor'/exp OR 'Breast Neoplasm':ab,ti OR 'Breast Tumor':ab,ti OR 'Breast Cancer':ab,ti OR 'Breast Carcinoma':ab,ti OR 'Mammary Cancer':ab,ti OR 'Mammary Carcinoma':ab,ti OR 'Mammary Neoplasm':ab,ti) AND ('positron emission tomography'/exp OR 'positron emission tomography':ab,ti OR 'PET':ab,ti) AND ('Bone metastasis':ab,ti OR 'Bone metastases':ab,ti)
Web of Science (527)	((AB=(Breast Neoplasms OR Breast Neoplasm OR Breast Tumor OR Breast Cancer OR Breast Carcinoma OR Mammary Cancer OR Mammary Carcinoma OR Mammary Neoplasm)) AND AB=(positron emission tomography OR PET)) AND AB=(Bone metastasis OR Bone metastases)
Cochrane Library(29)	(Breast Neoplasms OR Breast Neoplasm OR Breast Tumor OR Breast Cancer OR Breast Carcinoma OR Mammary Cancer OR Mammary Carcinoma OR Mammary Neoplasm):ti,ab,kw AND (Positron Emission Tomography OR PET):ti,ab,kw AND (Bone metastasis OR Bone metastases):ti,ab,kw

SUPPLEMENTARY TABLE 2. Technical aspects of included studies for [^{18}F]FDG PET/CT

Author	Year	Type of imaging test	Scanner Modality	Ligand dose	Time from injection to acquisition	Image analysis	TP, FP, FN, TN
Catalano *et al.*[15]	2015	PET/CT	Gemini TF; Philips, Best, the Netherlands	4.44 ± 1 MBq/kg	60 ± 10 minutes	Semiquantitative	TP:22, FP:1, FN:3, TN:83(PB)
Melsaether *et al.*[10]	2016	PET/CT	Biograph mCT imager (Siemens Healthcare, Knoxville, Tenn)	547.6 (484.7–566.1) MBq	45 minutes	Visual	TP:90, FN:13(LB)
Botsikas *et al.*[9]	2018	PET/CT	Biograph 64 scanner or a Biograph mCT scanner (Siemens Medical Solutions)	3.5 MBq/kg	185 minutes	Visual	TP:6, FP:0, FN:3, TN:71(PB) TP:18, FP:0, FN:8, TN:149(LB)
Sawicki *et al.*[11]	2016	PET/CT	Biograph mCT 128 (Siemens Healthcare GmbH, Erlangen, Germany)	257 ± 44 MBq	62.9 ± 13.1 minutes	Visual and semiquantitative	TP:61, FN:4(LB)
Balci *et al.*[17]	2012	PET/CT	Siemens Biograph 6 PET/CT system (Siemens AG, Munich, Germany)	370–550 MBq	60 minutes	Visual	TP:41, FP:0, FN:8, TN:113(PB)
Hahn *et al.*[18]	2011	PET/CT	BiographTM PET/CT system (Siemens Molecular Imaging, Hoffman Estates, IL, USA)	265 ± 37 MBq	60 minutes	Visual	TP:8, FP:0, FN:0, TN:21(PB) TP:67, FP:5, FN:3, TN:54(LB)
Manohar *et al.*[19]	2012	PET/CT	PET/CT scanner (Discovery STE 16; GE Healthcare, Milwaukee, Wisconsin, USA)	370–444 MBq	60 minutes	NA	TP:15, FP:0, FN:0, TN:20(LB)
Niikura *et al.*[25]	2011	PET/CT	Siemens ECAT HR with dedicated CT (Siemens/CTI, Knoxville, TN), GE Discovery ST 8-slice PET/CT, GE Discovery STE 16-slice PET/CT, GE Discovery RX 16-slice PET/CT, of GE VCT 64-slice PET/CT (General Electric Medical Systems, Milwaukee, WI)	555–740 MBq	60-90 minutes	Visual	TP:55, FP:7, FN:1, TN:162(LB)
Riegger *et al.*[22]	2012	PET/CT	Biograph mCT PET/CT system (Siemens Molecular Imaging)	280 ± 40 MBq	60 minutes	Visual	TP:7, FN:3(LB)
Rager *et al.*[23]	2018	PET/CT	Biograph 16-slice PET/CT scanner, Siemens Healthcare, Erlangen, Germany	370 MBq	60 minutes	Visual	TP:10, FP:0, FN:2, TN:13(PB) TP:43, FP:0, FN:48, TN:18(LB)

Author	Year	Type of imaging test	Scanner Modality	Ligand dose	Time from injection to acquisition	Image analysis	TP, FP, FN, TN
Demir *et al.*[20]	2014	PET/CT	16 slices multidetector spiral CT integrated PET scanner (G.E. Discovery 600)	296-555 MBq	60 minutes	NA	TP:218, FP:0, FN:44, TN:92(LB)
Hansen *et al.*[24]	2015	PET/CT	GE Healthcare Systems, Chicago, IL, USA	4 MBq/kg	60 and 180 minutes	Semiquantitative	TP:479, FN:9(LB)
Niikura *et al.*[25]	2016	PET/CT	Siemens Medical Solutions, Knoxville, TN	185–370 MBq	60 minutes	Semiquantitative	TP:7, FP:3, FN:0, TN:18(PB)
Shawky *et al.*[26]	2016	PET/CT	Siemens, Biograph mCT 128; Siemens Medical Solutions, Knoxville, USA	370–550 MBq	60 minutes	Visual and semiquantitative	TP:11, FP:0, FN:1, TN:18(LB)
Teke *et al.*[27]	2020	PET/CT	Biograph 6 PET/CT scanner (CTI/Siemens, Knoxville, USA)	370–555 MBq	60 minutes	Visual	TP:141, FP:2, FN:10, TN:343(LB)

FN = false positive; FP = false positive; LB = lesion-based; NA = not available; PB = patient-based; TP = true positive; TN =rue negative

SUPPLEMENTARY TABLE 3. Technical aspects of included studies for [^{18}F]FDG PET/MRI

Author	Year	Type of imaging test	Scanner Modality	Ligand dose	Time from injection to acquisition	Image analysis	TP, FP, FN, TN
Catalano *et al.*[15]	2015	PET/MRI	Biograph mMR imager (Siemens Healthcare, Erlangen, Germany)	4.44 ± 1 MBq/kg	125.8 ± 25.74 minutes	Semiquantitative	TP:25, FP:0, FN:0, TN:84(PB)
Bruckmann *et al.*[21]	2021	PET/MRI	3.0-Tesla Biograph mMR scanner (Siemens, Healthineers)	254.4 ± 43.6 MBq	64 ± 17 minutes	Visual	TP:7, FP:0, FN:0, TN:147(PB) TP:41, FP:0, FN:0, TN:4(LB)
Melsaether *et al.*[10]	2016	PET/MRI	3-T Biograph MR system (Siemens Healthcare, Knoxville, Tenn)	547.6 (484.7–566.1) MBq	167 ± 36 minutes	Visual	TP:105, FN:2(LB)
Botsikas *et al.*[9]	2018	PET/MRI	Philips Ingenuity TF PET/MR (Philips Healthcare)	3.5 MBq/kg	90 minutes	Visual	TP:8, FP:2, FN:1, TN:69(PB) TP:24, FP:7, FN:2, TN:142(LB)
Sawicki *et al.*[11]	2016	PET/MRI	3 Tesla PET/MRI scanner (Biograph mMR, Siemens Healthcare GmbH, Erlangen, Germany)	257 ± 44 MBq	124.8 ± 28.9 minutes	Visual and semiquantitative	TP:65, FN:0(LB)

FN = false positive; FP = false positive; LB = lesion-based; NA = not available; PB = patient-based; TP = true positive; TN =rue negative

UPPLEMENTARY TABLE 4. Complementary role of PET/CT and PET/MRI in identifying bone metastasis in patients with negative imaging results from the other test

Author, year	Patients or lesions with negative PET/CT		Patients or lesions with negative PET/MRI		PET/MRI Detection rate			PET/CT Detection rate		
	Total patients or lesions	True positive patients or lesions detected by PET/MRI	Total patients or lesions	True positive patients or lesions detected by PET/CT	Detected by MRI alone	Detected by PET alone	Detected by both PET and MRI	Detected by CT alone	Detected by PET alone	Detected by both PET and CT
Sawicki *et al.* 2016[11]	21	5	16	0	129/134	NA	NA	100/134	NA	NA
Botsikas *et al.* 2018[9]	74	2	70	0	NA	NA	NA	NA	NA	NA
Melsaether *et al.* 2016[10]	NA	NA	NA	NA	NA	NA	NA	NA	NA	NA
Catalano *et al.* 2015[15]	3	3	NA	NA	51/141	5/141	85/141	2/90	37/90	51/90
Balci *et al.* 2012[17]	NA	NA	NA	NA	NA	NA	NA	NA	NA	NA

Author, year	Patients or lesions with negative PET/CT		Patients or lesions with negative PET/MRI		PET/MRI Detection rate			PET/CT Detection rate		
	Total patients or lesions	True positive patients or lesions detected by PET/MRI	Total patients or lesions	True positive patients or lesions detected by PET/CT	Detected by MRI alone	Detected by PET alone	Detected by both PET and MRI	Detected by CT alone	Detected by PET alone	Detected by both PET and CT
Hahn *et al.* 2011[18]	NA	NA	NA	NA	NA	NA	NA	54/70	NA	61/70
Niikura *et al.* 2011[25]	NA	NA	NA	NA	NA	NA	NA	NA	NA	NA
Manohar *et al.* 2012[19]	NA	NA	NA	NA	NA	NA	NA	NA	NA	NA
Riegger *et al.* 2012[22]	NA	NA	NA	NA	NA	NA	NA	NA	NA	NA
Rager *et al.* 2018[23]	NA	NA	NA	NA	NA	NA	NA	NA	NA	NA
Demir *et al.* 2014[20]	NA	NA	NA	NA	NA	NA	NA	NA	NA	NA
Hansen *et al.* 2015[24]	NA	NA	NA	NA	NA	NA	NA	NA	NA	NA
Niikura *et al.* 2016[21]	NA	NA	NA	NA	NA	NA	NA	NA	NA	NA
Shawky *et al.* 2016[26]	NA	NA	NA	NA	NA	NA	NA	10/30	NA	NA
Teke *et al.* 2020[27]	NA	NA	NA	NA	NA	NA	NA	NA	NA	NA
Total	98	10	86	0	180/275	5/141	85/141	166/324	37/90	112/160

NA = not available

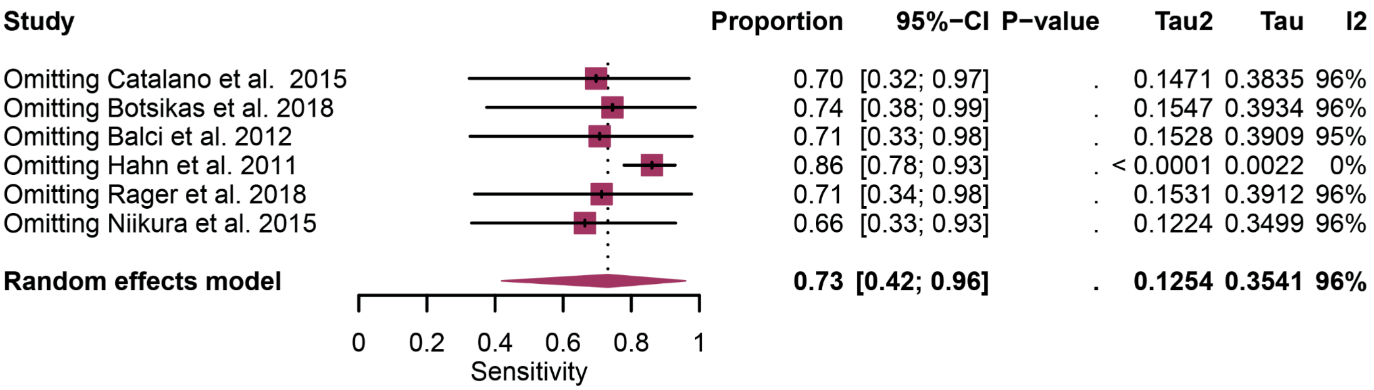

SUPPLEMENTARY FIGURE 1. Sensitivity analysis for the pooled sensitivity of [18F]FDG PET/CT in bone metastasis of breast cancer patients on a patient-based analysis.[9,15,17,18,21,23]

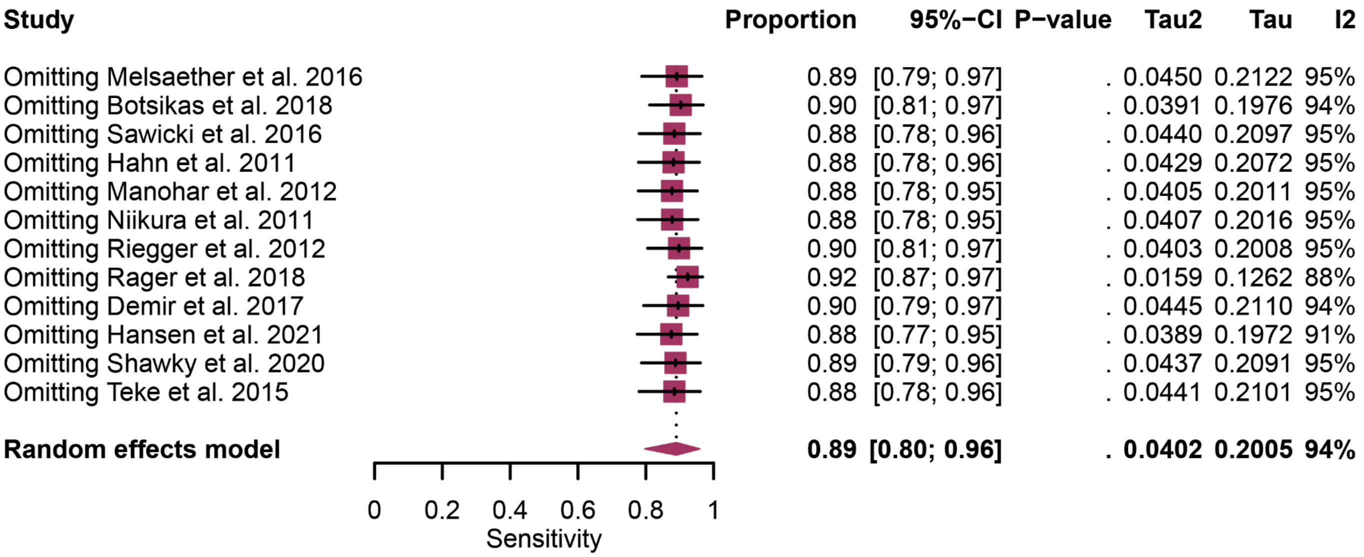

SUPPLEMENTARY FIGURE 2. Sensitivity analysis for the pooled sensitivity of [18F]FDG PET/CT in bone metastasis of breast cancer patients on a lesion-based analysis.[9-11,18-20,22-27]

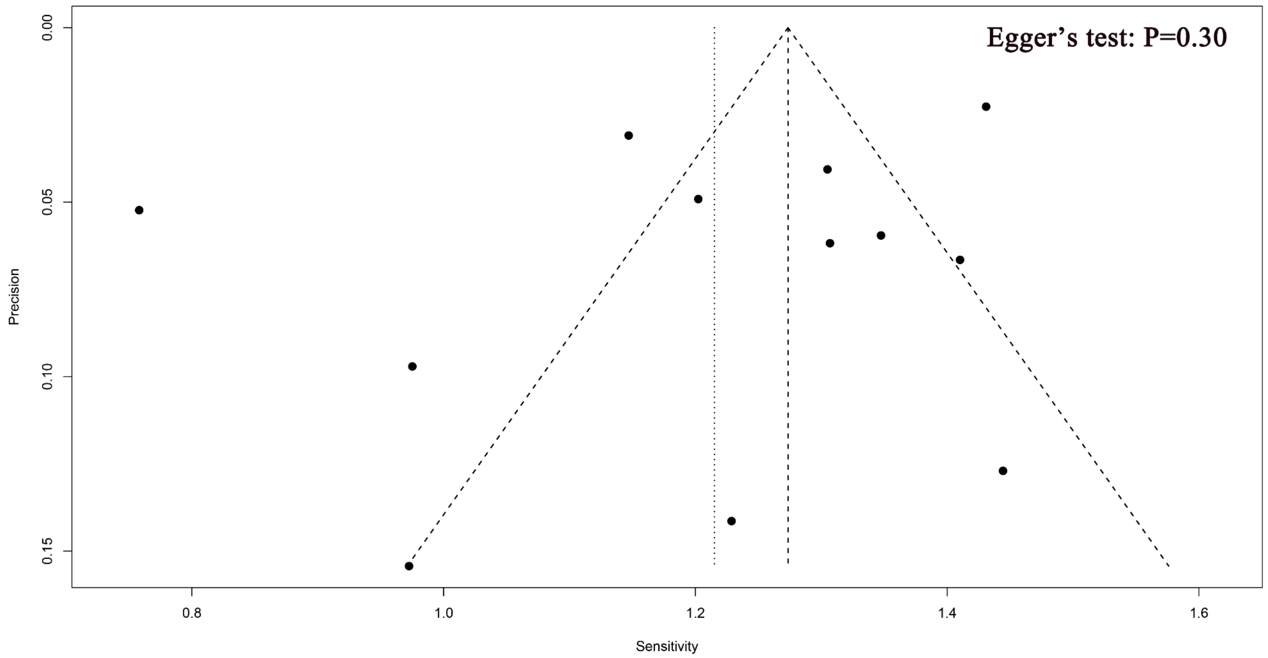

SUPPLEMENTARY FIGURE 3. The funnel plot and Egger's test revealed no evidence of publication bias for [18F]FDG PET/CT in lesion-based analysis.

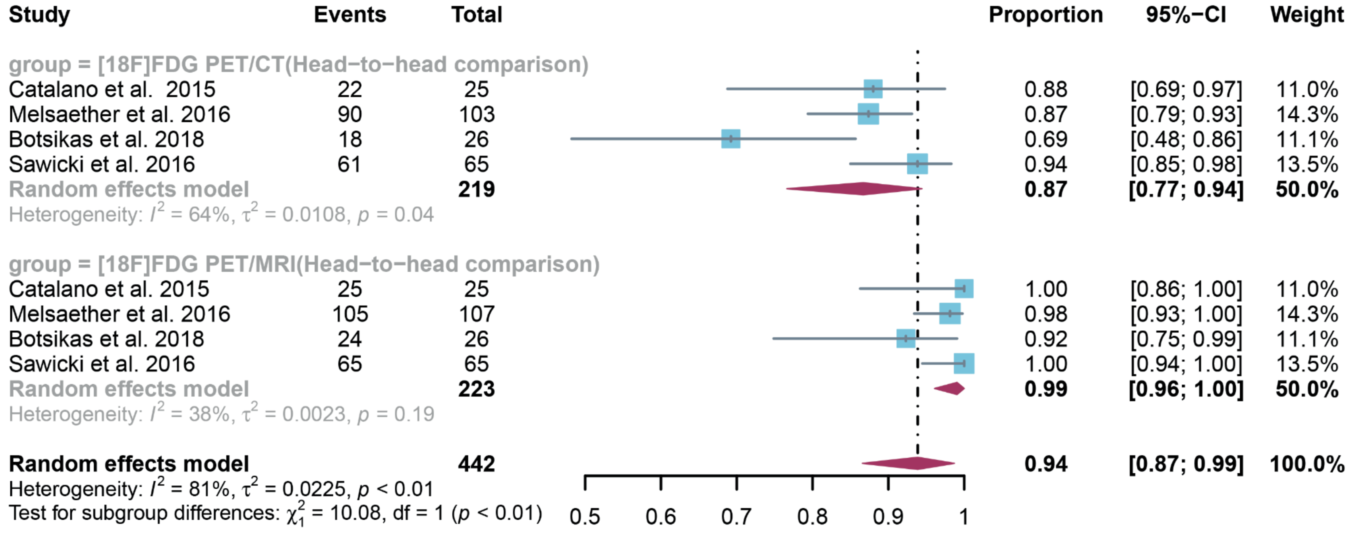

SUPPLEMENTARY FIGURE 4. Forest plot showing the head-to-head comparison of pooled sensitivities for [18F]FDG PET/CT and [18F]FDG PET/MRI in detecting bone metastases in breast cancer patients. The plot displays individual study estimates (squares) with corresponding 95% confidence intervals (horizontal lines) and the pooled sensitivity estimate (diamond) for both modalities. The size of the squares represents the relative weight of each study in the meta-analysis.[9-11,15]

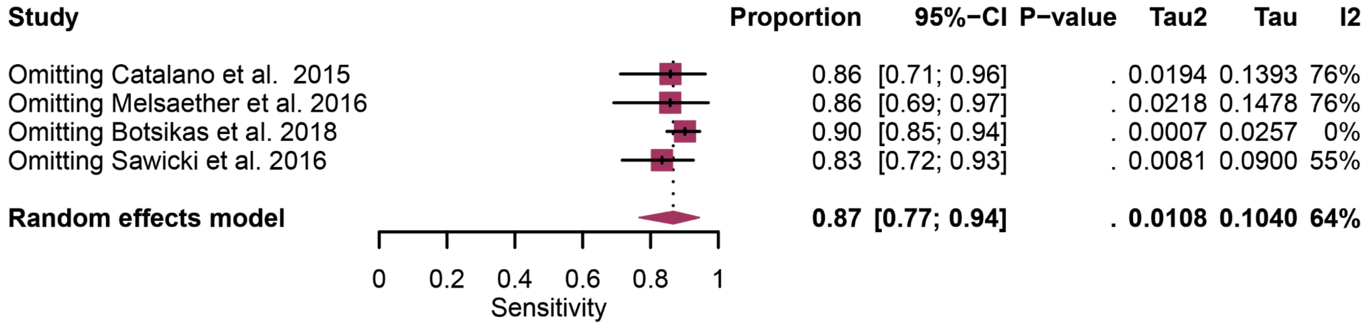

SUPPLEMENTARY FIGURE 5. Sensitivity analysis for the pooled sensitivity of [18F]FDG PET/CT in bone metastasis of breast cancer patients on a head-to-head comparison.[9-11,15]

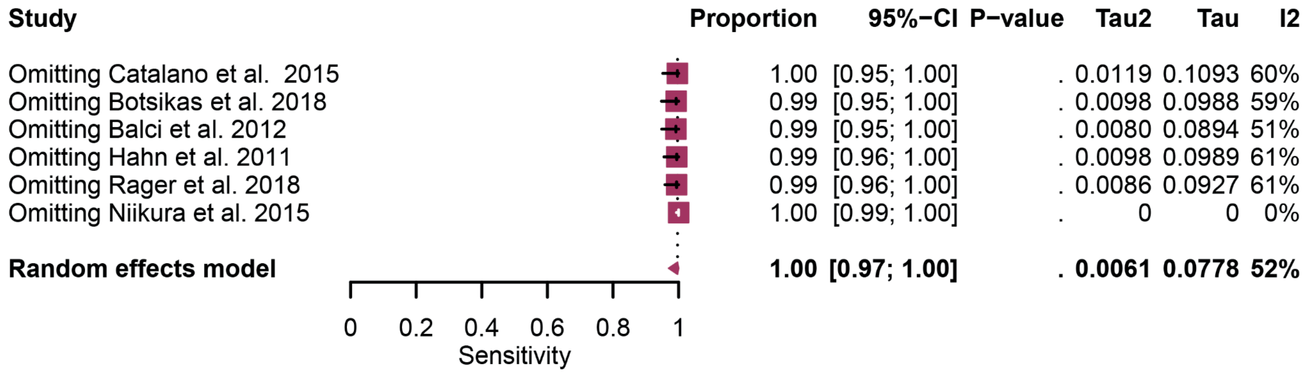

SUPPLEMENTARY FIGURE 6. Sensitivity analysis for the pooled specificity of [18F]FDG PET/CT in bone metastasis of breast cancer patients on a patient-based analysis.[9,15,17,18,21,23]

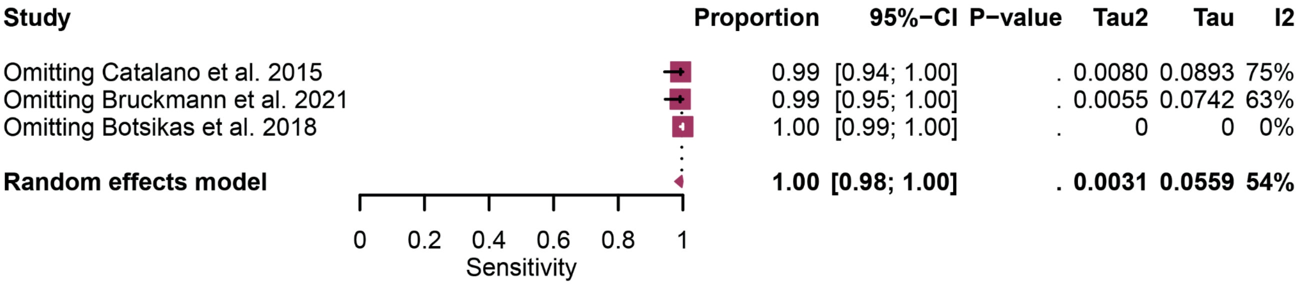

SUPPLEMENTARY FIGURE 7. Sensitivity analysis for the pooled specificity of [18F]FDG PET/MRI in bone metastasis of breast cancer patients on a patient-based analysis.[9,15,21]

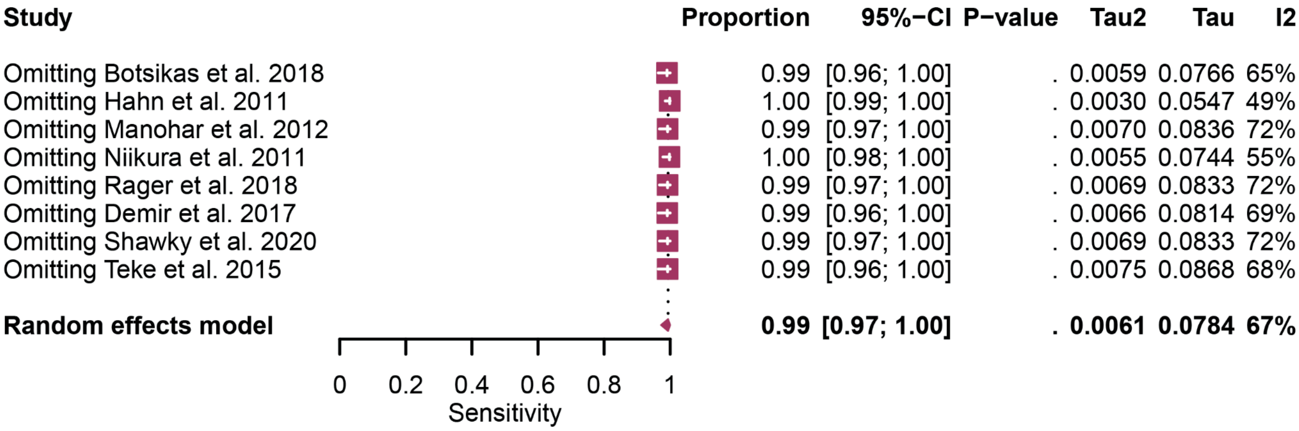

SUPPLEMENTARY FIGURE 8. Sensitivity analysis for the pooled specificity of [18F]FDG PET/CT in bone metastasis of breast cancer patients on a lesion-based analysis.[9,18-20,23,25-27]

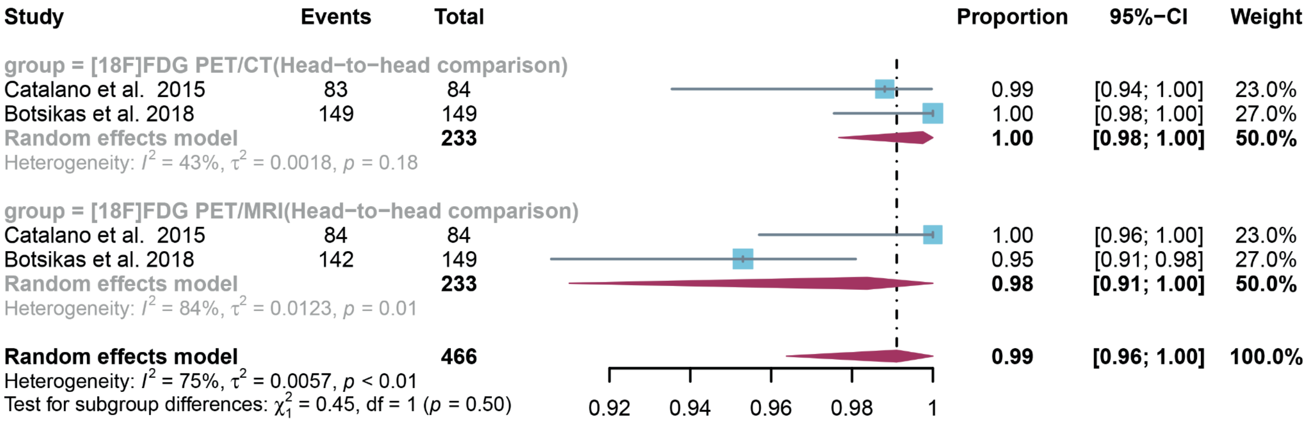

SUPPLEMENTARY FIGURE 9. Forest plot showing the head-to-head comparison of pooled specificities for [18F]FDG PET/CT and [18F]FDG PET/MRI in detecting bone metastases in breast cancer patients. The plot displays individual study estimates (squares) with corresponding 95% confidence intervals (horizontal lines) and the pooled specificity estimate (diamond) for both modalities. The size of the squares represents the relative weight of each study in the meta-analysis.[9,15]
